# Gait Speed and All‐Cause Mortality in Whole‐Spectrum Chronic Kidney Disease: A Systematic Review and Meta‐Analysis Included 6217 Participants

**DOI:** 10.1002/jcsm.13739

**Published:** 2025-02-24

**Authors:** Fan Zhang, Hui Wang, Yan Bai, Liuyan Huang, Yifei Zhong, Yi Li

**Affiliations:** ^1^ Department of Nephrology A Longhua Hospital Shanghai University of Traditional Chinese Medicine Shanghai China; ^2^ Department of Anorectal Longhua Hospital Shanghai University of Traditional Chinese Medicine Shanghai China

**Keywords:** chronic kidney disease, gait speed, meta‐analysis, mortality, systematic review

## Abstract

**Background:**

The quantitative relationship between gait speed and mortality risk in patients with chronic kidney disease remains unclear. This study aimed to conduct a meta‐analysis to estimate the risk of mortality associated with gait speed in chronic kidney disease (CKD) patients.

**Methods:**

Relevant studies published were identified through literature searches using Embase, PubMed and Web of Science. Prospective cohort studies of adult CKD patients that examined the relationship between gait speed and mortality were included. Random effects meta‐analyses based on restricted maximum likelihood to were used to calculate relative risk (RR) and 95% confidence interval (95% CI). The results of meta‐analyses were assessed using Grading of Recommendations, Assessment, Development and Evaluation framework.

**Results:**

Seventeen prospective cohort studies involving 6217 CKD patients (mean age range: 51.6–81.85 years; 44.3%–84% male) were included. Pooled analysis of 12 studies (*n* = 4233) showed that lower gait speed was associated with a higher risk of all‐cause mortality compared to higher gait speed (RR = 2.138; 95% CI: 1.794–2.548; *p* < 0.001; *I* ^2^ = 16.0%; high‐certainty evidence) in CKD patients. Dose–response meta‐analysis of 6 studies (*n* = 1650) revealed that each 0.1 m/s increase in gait speed was associated with a 25.7% lower risk of all‐cause mortality (RR = 0.743; 95% CI: 0.580–0.955; *p* = 0.018; *I* ^2^ = 45.0%; high‐certainty evidence).

**Conclusions:**

Slower gait speed is a strong predictor of all‐cause mortality in CKD patients, including those undergoing dialysis or kidney transplantation. Gait speed assessment should be incorporated into routine clinical evaluations to identify high‐risk patients and guide interventions aimed at improving physical function and survival outcomes.

**Trial Registration:** PROSPERO registration number: CRD42022340135

## 1 Introduction

Chronic kidney disease (CKD) is a global public health problem characterized by high incidence, low awareness, poor prognosis and high medical costs [1]. Recent estimates suggest that the global prevalence of CKD is approximately 9.5%, affecting over 740 million people worldwide [2]. In high‐income countries, CKD has become the ninth leading cause of death, with its incidence increasing yearly and trending toward younger population [3]. The global loss of life expectancy due to CKD is expected to double by 2040, representing a formidable challenge for healthcare and health systems [4].

Gait speed, defined as the speed at which a person habitually walks, has emerged as a significant predictor of health outcomes in various populations [5]. In healthy adults, gait speed has been associated with reduced risks of all‐cause mortality and cognitive decline [6, 7]. A recent UK Biobank–based cohort study showed that participants with faster gait speeds had a 47% lower risk of developing CKD compared to those with slower gait speeds [8]. These findings suggest that gait speed may be a valuable indicator of overall health status and future risk in CKD patients.

In the context of CKD, physical function measures like gait speed are particularly relevant due to the high prevalence of frailty and sarcopenia in this population [9]. CKD patients often experience a decline in physical function as their disease progresses, which can impact their quality of life and clinical outcomes [10]. Studenski et al. demonstrated in a pooled analysis of nine cohort studies that gait speed was associated with survival in older adults, with each 0.1 m/s increase in gait speed corresponding to a 12% decrease in mortality risk [11].

Although previous studies have explored the relationship between gait speed and mortality in CKD patients, a comprehensive quantitative analysis is lacking. A recent meta‐analysis by Yang, He, and Li reported an association between gait speed and all‐cause mortality in CKD [12]. However, our study aims to build upon this work by including a larger number of recent published studies, employing more rigorous inclusion criteria and methodological assessments, conducting dose–response analyses and performing more extensive subgroup analyses to explore potential sources of heterogeneity. Additionally, we will use the Grading of Recommendations, Assessment, Development and Evaluation (GRADE) framework to assess the certainty of evidence, providing a more comprehensive evaluation of the relationship between gait speed and mortality risk in CKD patients across the disease spectrum.

The primary objective of this systematic review and meta‐analysis is to clarify the quantitative relationship between gait speed and all‐cause mortality risk in adults with CKD, including patients at various stages of the disease from non‐dialysis to those receiving renal replacement therapy.

## 2 Methods

The review protocol has been registered with PROSPERO (CRD42022340135) and reported following the Preferred Reporting Items for Systematic Reviews and Meta‐Analyses (PRISMA) 2020 statement (Table S1) [13] and Meta‐analysis of Observational Studies in Epidemiology (MOOSE) [14].

### 2.1 Search Strategy

We searched PubMed, Embase and Web of Science for English‐language reports on the association between gait speed and all‐cause mortality in patients with CKD from inception to 2 January 2024 and updated search in September 2024. Details of the search strategy are provided in Table S2. In addition, reference lists of identified articles were manually searched for relevant articles.

### 2.2 Study Selection

Two researchers (F.Z. and Y.B.) independently screened the literature. Studies were included if they were (1) prospective cohort studies. We did not set a minimum follow‐up time as an inclusion criterion. This is because CKD patients have a higher risk of death, and meaningful results may be observed even with short‐term follow‐up [15]. However, we considered the effect of follow‐up time (≤ 36 months vs. > 36 months) in the subgroup analysis; (2) participants were adult CKD patients (≥ 18 years of age), including non‐dialysis, peritoneal dialysis, haemodialysis and kidney transplantation recipients; (3) gait speed (measured by timed walking speed test or self‐reported) was considered as an exposure factor; (4) multivariable adjusted effect size (odds ratio [OR] or hazard ratio [HR] or relative risk [RR]) and 95% confidence interval (95% CI) were reported for the risk of all‐cause mortality of CKD for different gait speed categories; and (5) consider all‐cause mortality during follow‐up as an outcome. Conference abstracts, clinical trials, cross‐sectional studies or reviews were excluded. If multiple articles based on the same cohort were published, we chose the one with the longest follow‐up or the largest sample size.

### 2.3 Duplicate Cohort Description

Both Roshanravan et al. [10, 16] are from the Seattle Kidney Study; in addition, Kutner et al. [17] and Johansen et al. [18] are from A Cohort To Investigate the Value of Exercise/Analyses Designed to Investigate be the Paradox of Obesity and Survival in ESRD (ACTIVE/ADIPOSE), both of which chose larger sample sizes and closer years, therefore excluding Roshanravan et al. [16] and Kutner et al. [17].

### 2.4 Data Extraction and Quality Assessment

Two researchers (F.Z. and H.W.) independently extracted data, including first author, year of publication, country, sample size, number of cases, length of follow‐up, gender, mean age of study participants at baseline, gait speed measurements, level of walking speed, effect sizes corresponding to mortality and 95% CIs (adjusted for up to confounders) and adjustment variables. The quality of each study was assessed using the Newcastle–Ottawa Scale. Two researchers (F.Z. and H.W.) independently performed the quality assessment, and disagreements were resolved by a third researcher (Y.F.Z.).

### 2.5 Data Synthesis and Analysis

Due to a low incidence of the outcome (all‐cause mortality), we used RR as a uniform effect size across all studies and assumed that HR and OR reported in the original study approximated the RR [19]. Effect sizes and corresponding 95% CIs were extracted from each study, and the pooled RR (95% CI) was then calculated using a random effects model and restricted maximum likelihood (REML) adjustment. According to priori protocols, we first performed a paired meta‐analysis to estimate the RR and 95% CI corresponding to all‐cause mortality risk of CKD for different gait speed categories compared to the highest speed used as a reference. For studies where the reference category was not the highest, we recalculated the effect sizes and 95% CIs, assuming that the highest category was the reference category according to Hamling's method [20]. In the further analyses, we summarized the risk estimates using unit increments (0.1 m/s per acceleration). When studies presented multiple statistical risk‐adjustment models, only the effect sizes associated with the statistical model containing the most additional covariates were considered. In addition, 95% prediction intervals were calculated to forecast the actual effects' range [21].

Heterogeneity was tested using Cochran *Q* and *I* ^2^ statistics [22]. *Q* statistic *p* < 0.10 was considered statistically significant, and *I* ^2^ ≈ 25%, 50% and 75% were considered low, intermediate and high heterogeneity, respectively. In addition, we performed meta‐regression analyses to identify sources of heterogeneity.

We performed the following subgroup analyses.

1. Age (< 60 vs. > = 60 years): 60 years is usually considered a starting age for the elderly, and the prevalence of CKD is significantly higher in people older than 60 years.
2. Duration of follow‐up (< = 36 vs. > 36 months): 36 months represents the mid‐term follow‐up. This allowed us to compare differences in short/intermediate and long‐term follow‐up outcomes.
3. Gender predominance (< 50% vs. ≥ 50% male): to assess the potential impact of gender composition on outcomes.
4. Disease stage (non‐dialysis vs. renal replacement treatment): to reflect the different severity and treatment stages of CKD.
5. Region (North America vs. Europe vs. Asian): to assess the impact of geographic and possible racial differences on outcomes.

We also performed two sensitivity analyses, including omitting one study at a time to examine each study's effect on the pooled results and only studies using 0.8 m/s as the cut‐off point for walking speed. Funnel plots graphically assessed publication bias [23] and were further evaluated by Egger's test, with *p* < 0.05 indicating potential publication bias. Trim‐and‐fill method was used to correct for the identified publication bias. All analyses were performed using *R* software (Version 4.2.2) [24]. A two‐tailed *p* < 0.05 was considered statistically significant if not otherwise stated.

### 2.6 Grading of Evidence Assessment

Two independent reviewers (Y.B. and L.Y.H.) assessed the cumulative evidence for each outcome using the GRADE framework. The certainty of evidence was rated as high, medium, low, and very low by the GRADE tool [25].

## 3 Results

### 3.1 Literature Search and Study Selection Process

**FIGURE 1:**
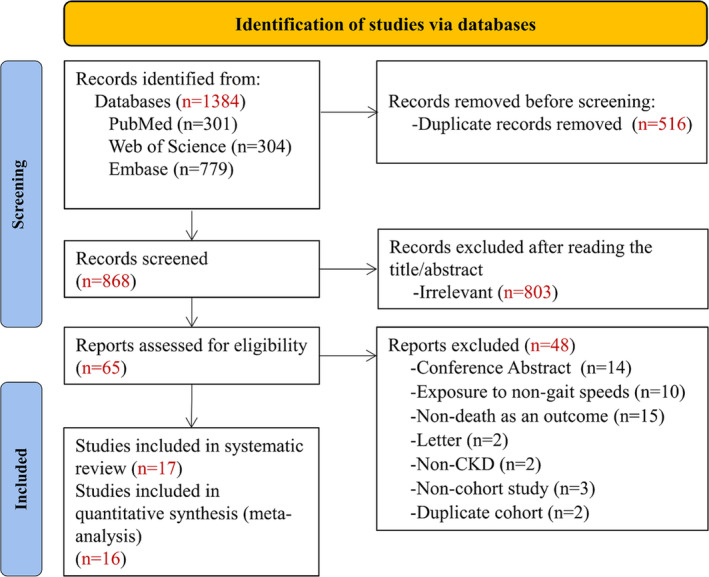
Preferred Reporting Items for Systematic Reviews and Meta‐Analyses flow diagram.

Figure 1 shows the systematic search and study screening process. After excluding 516 duplicate records and an additional 803 records that did not meet the inclusion criteria, we read the full text of 65 records; of these, 17 cohort studies were deemed to meet the inclusion criteria [10, 16, 18, 26, 27, 28, 29, 30, 31, 32, 33, 34, 35, 36, 37, 38, 39]. Table S3 lists the studies that were excluded by reading full text (*n* = 48), with reasons for exclusion.

### 3.2 Characteristics of Cohort Studies

Seventeen prospective cohort studies [10, 16, 18, 26, 27, 28, 29, 30, 31, 32, 33, 34, 35, 36, 37, 38, 39] involving 936 cases among 6217 participants (two of these studies did not report deaths) (Table 1). One study reported all‐cause mortality risk corresponding to each 1‐point decrease in gait speed based on the Short Physical Performance Battery, which was not included in the meta‐analysis [34]. The included cohort studies were conducted in the United States (*n* = 4), Canada (*n* = 1), the United Kingdom (n = 1), Spain (*n* = 1), China (*n* = 3), Iran (*n* = 1), Japan (*n* = 4), South Korea (*n* = 1), and Brazil (*n* = 1) and were published between 2013 and 2023. The cohort studies had a follow‐up period ranging from 12 to 62.4 months. The risk assessment of the included studies according to the Newcastle–Ottawa Scale tool is shown in Table S4.

**TABLE 1:** Characteristics of cohort studies included in the meta‐analysis.

Author, year	Country	Participants	CKD stage	Age	% Male	Duration of follow‐up	Dead	Assessing of walking speed	Walking speed categories	Relative risk (95% CI)	Adjustments
Mayrink Ivo et al., 2023 [31]	Brazil	97	Haemodialysis	50.93 ± 14.10	71.13	32 months	29	A distance of 3 min	Per 0.1 m/s increase	1.18 (0.18–7.62)	None
Roshanravan et al., 2013 [10]	USA	385	Pre‐dialysis	61 ± 13	84	36 months	50	A distance of 4 m	Per 0.1 m/s slower	1.26 (1.09–1.47)	Age, sex, race, study site, smoking, BMI, diabetes, prevalent coronary artery disease and eGFR
									> 0.8 m/s	Ref.	
									< = 0.8 m/s	2.45 (1.09–5.54)	
Clarke et al., 2019 [28]	UK	450	Pre‐dialysis	62 ± 20^a^	57	43 months	74	Self‐reported	< 3 mph (< 1.3 m/s)	Ref.	Age, gender, ethnicity, eGFR, haemoglobin, diabetes mellitus, hypertension and ischaemic heart disease
									> 3 mph (> 1.3 m/s)	0.37 (0.20–0.71)	
Tabibi et al., 2020 [36]	Iran	79	Peritoneal dialysis	53.3 ± 3.4^b^	44.3	Not reported	19	A distance of 4 m	< = median	0.5 (0.1–2.2)	Total dialysis adequacy
									> median	Ref.	
Nakano et al., 2023 [33]	Japan	251	Pre‐dialysis	75 ± 9^a^	65	5.2 years	22	Not reported	> 0.8 m/s	Ref.	Age, gender, BMI, diabetes mellitus and CVD history, CKD stage and serum albumin
									< = 0.8 m/s	0.88 (0.40–1.92)	
Johansen et al., 2019 [18]	USA	771	Haemodialysis	57.2 ± 14.3	59.2	3.8 years	204	A distance of 4 m	< = 0.65 m/s	2.31 (1.70–3.15)	Age, sex, race, ethnicity, dialysis vintage, body mass index, diabetes, atherosclerotic heart disease, heart failure, dialysis via a catheter and serum albumin concentration
									> 0.65 m/s	Ref.	
Lee et al., 2020 [39]	Korea	277	Haemodialysis	61.7 ± 13.0^b^	66.1	25.3 months	19	A distance of 4 m	< = 0.8 m/s	1.92 (0.69–5.31)	Age, sex, previous history of cardiovascular disease, serum albumin and Charlson comorbidity score
									> 0.8 m/s	Ref.	
Lin et al., 2020 [40]	China	126	Haemodialysis	63.2 ± 13.0	51.6	3 years	26	A distance of 6 m	Per 0.1 m/s increase	0.61 (0.31–1.20)	Age, sex, haemoglobin, phosphorus, albumin and Kt/V
McAdams‐DeMarco et al., 2017 [32]	USA	663	Kidney transplant recipients	53.0 ± 13.9	62	3.1 years	Not reported	A distance of 15 ft	< 0.6 m/s	2.43 (1.17–5.03)	Age, sex, race, donor type and Charlson Comorbidity Index
									> = 0.6 m/s	Ref.	
Chen et al., 2023 [27]	China	923	Haemodialysis	61.3 ± 12.7	61.2	14 months	79	A distance of 4 m	Per 0.1 m/s increase	0.40 (0.18–0.92)	Age, sex, BMI, IPAQ, smoking, falling history, depression, malnutrition, Charlson comorbidity index, haemoglobin, albumin and Kt/V
Nastasi et al., 2018 [34]^c^	USA	719	Kidney transplant recipients	51.6 ± 14.2	62.3	2.0 years	Not reported	A distance of 8 ft	Per 1‐point decrease	1.21 (0.89–1.65)	Age, sex, race, BMI, years on dialysis, cause of ESRD, donor type, cardiovascular disease, lung disease and diabetes
Yoshikoshi et al., 2023 [38]^d^	Japan	496	Haemodialysis	65.5 ± 12.3^a^	59.1	Not reported	186	A distance of 16 m	Low reserved gait capacity	Ref.	Age, sex, BMI, haemodialysis vintage, comorbidity index score, serum albumin, serum creatinine, serum haemoglobin, CRP and usual gait speed
									Moderate reserved gait capacity	0.66 (0.46–0.94)	
									High reserved gait capacity	0.44 (0.30–0.65)	
Kamijo et al., 2018 [29]	Japan	119	Peritoneal dialysis	66.8 ± 13.2	70.6	19.64 months	7	A distance of 10 m	Per 0.1 m/s increase	19.3 (0.82–454.1)	Age, gender, Charlson comorbidity index, body mass index, normalized protein equivalent nitrogen appearance and Clinical Frailty Scale
Sánchez‐Tocino et al., 2022 [35]	Spain	60	Haemodialysis	81.85 ± 5.58	68.3	24 months	30	A distance of 4 m	< = 0.8 m/s	2.35 (0.78–7.08)	Age, cardiovascular disease
									> 0.8 m/s	Ref.	
Li et al., 2021 [30]	China	150	Haemodialysis	69 ± 8^a^	48.7	12 months	15	Not reported	< 0.8 m/s	5.56 (1.41–22.00)	Age, gender, albumin, medical history of diabetes mellitus and coronary heart disease, low‐density lipoprotein cholesterol, smoking status and urea reduction rate
									> = 0.8 m/s	Ref.	
Brar et al., 2019 [26]	Canada	109	Dialysis	55.6 ± 16.8^b^	67	3.3 years	38	A distance of 4 m	< 0.8 m/s	1.28 (0.60–2.73)	Age, sex, albumin, haemoglobin and comorbidity count
									> = 0.8 m/s	Ref.	
Yamamoto et al., 2021 [37]	Japan	542	Haemodialysis	65.3 ± 12.1	60.3	3 years	138	A distance of 4 m	< 1 m/s	2.29 (1.32–3.98)	Age, sex, body mass index, time on haemodialysis, comorbidity index and serum albumin
									> = 1 m/s	Ref.	

Abbreviations: BMI, body mass index; CKD, chronic kidney disease; CRP, C‐reactive protein; CVD, cardiovascular disease; eGFR, estimated glomerular filtration rate; ESRD, end‐stage renal disease; IPAQ, International Physical Activity Questionnaire.

^a^ Mean ± SD was estimated using the method of Luo et al. [41] and Wan et al. [42]. Calculation results are available on the web (https://www.math.hkbu.edu.hk/~tongt/papers/median2mean.html).

^b^ Data were reported in subgroups (e.g., peritoneal dialysis and haemodialysis), and we combined them according to the methodology recommended by the Cochrane [43].

^c^ In this study, walking speed was one of the dimensions used to assess Short Physical Performance Battery, and the authors categorized walking speed into 4 levels on a score of 1–4 and reported the risk of CKD death for each 1‐point decrease in walking speed.

^d^ In this study, the authors calculated reserved gait capacity (an individual's usual gait speed/maximum gait speed) and grouped the results in tertiles.

### 3.3 Gait Speed Assessment

Twelve and six cohort studies reported the association between gait speed categories compared to reference, gait speed per unit increase and risk of death, respectively (one study reported both [10]). Sixteen cohort studies measured gait speed through a timed walking speed test, whereas another used a self‐report questionnaire. Six studies used 0.8 m/s as a threshold for assessing low walking speed, which is the most common walking speed cut‐off. The walking distance tests used in the included studies ranged from 3 to 16 m. See Table 1 for more information.

### 3.4 Categorical Analyses

**FIGURE 2:**
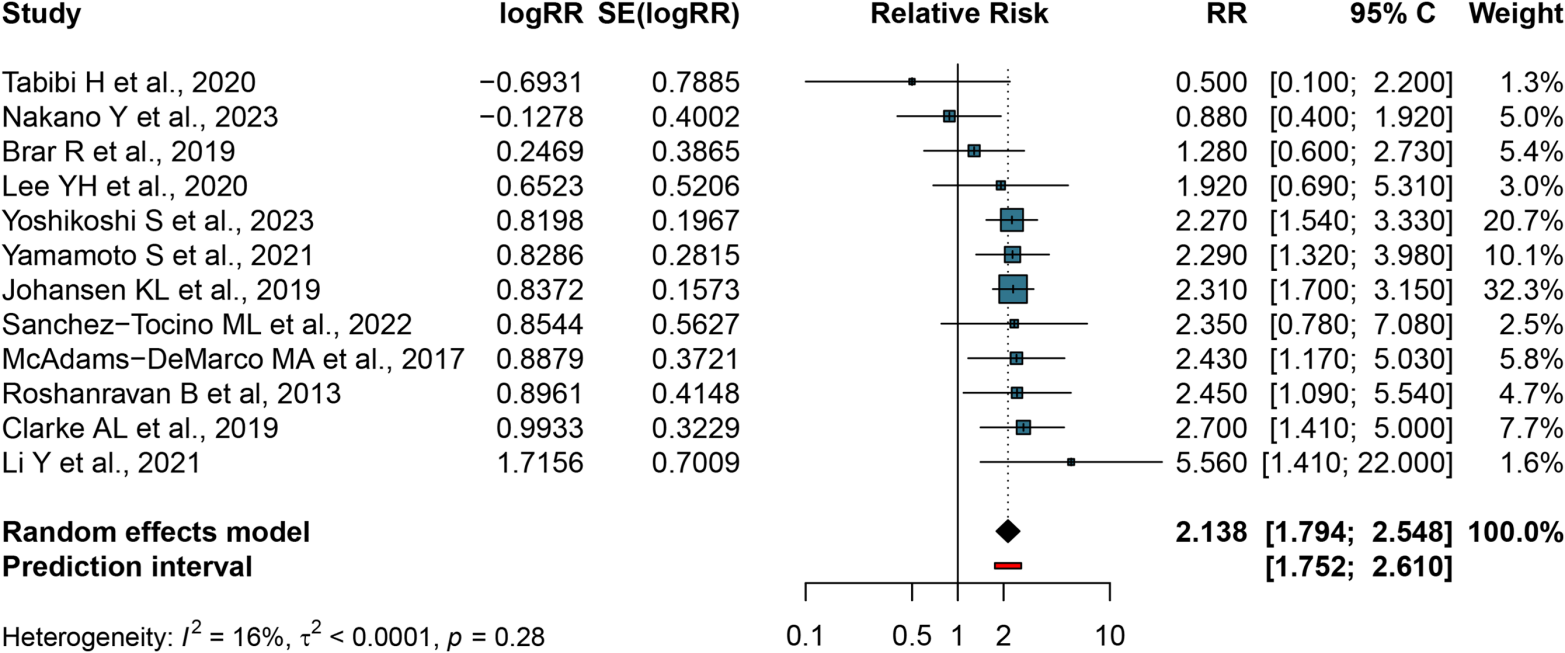
Forest plot for multivariable analysis assessing the association between gait speed and mortality risk. 95% CI, 95% confidence interval; RR, relative risk.

We performed categorical analyses to summarize the risk of all‐cause mortality for higher vs. lower gait speed, with 4233 participants and 795 cases. Compared with higher gait speed, the all‐cause mortality risk for lower was 2.138 (95% CI: 1.794–2.548; *I* ^2^ = 16.0%; *P*_for heterogeneity_ = 0.28; 95% prediction intervals: 1.752–2.610) (Figure 2). We rate the evidence certainty of this outcome as high (Table S5). Negative effects of similar magnitude and significance were found for leave‐one‐out sensitivity analysis (Figure S1). No publication bias was observed by the funnel plot (Figure S2) and Egger's test (*p* = 0.356). Sensitivity analysis illustrates the robustness of results (Figure S3).

In addition, the combined results of six studies that used 0.8 as a threshold showed that participants with ≥ 0.8 m/s had a 76.9% increased risk of death compared with CKD patients with walking speeds below 0.8 m/s (RR = 1.769; 95% CI: 1.127–2.778; *I* ^2^ = 31.2%; *P*_for heterogeneity_ = 0.20; 95% prediction intervals: 0.617–5.076) (Figure S4).

Another study [34] also supported the above findings but was excluded from the meta‐analysis because it reported point changes on the Short Physical Performance Battery rather than the actual speeds reported in other studies. Nastasi et al. [34] included 719 kidney transplant recipients and assessed their Short Physical Performance Battery performance at admission. During follow‐up, the 5‐year post‐transplant mortality rate was 20.6% in impaired recipients compared with 4.5% in unimpaired recipients. A 1‐point decrease in walking speed (worse function) was associated with an adjusted 1.21‐fold (95% CI: 0.89–1.65, *p* = 0.22) increase in mortality risk after kidney transplant.

**FIGURE 3:**
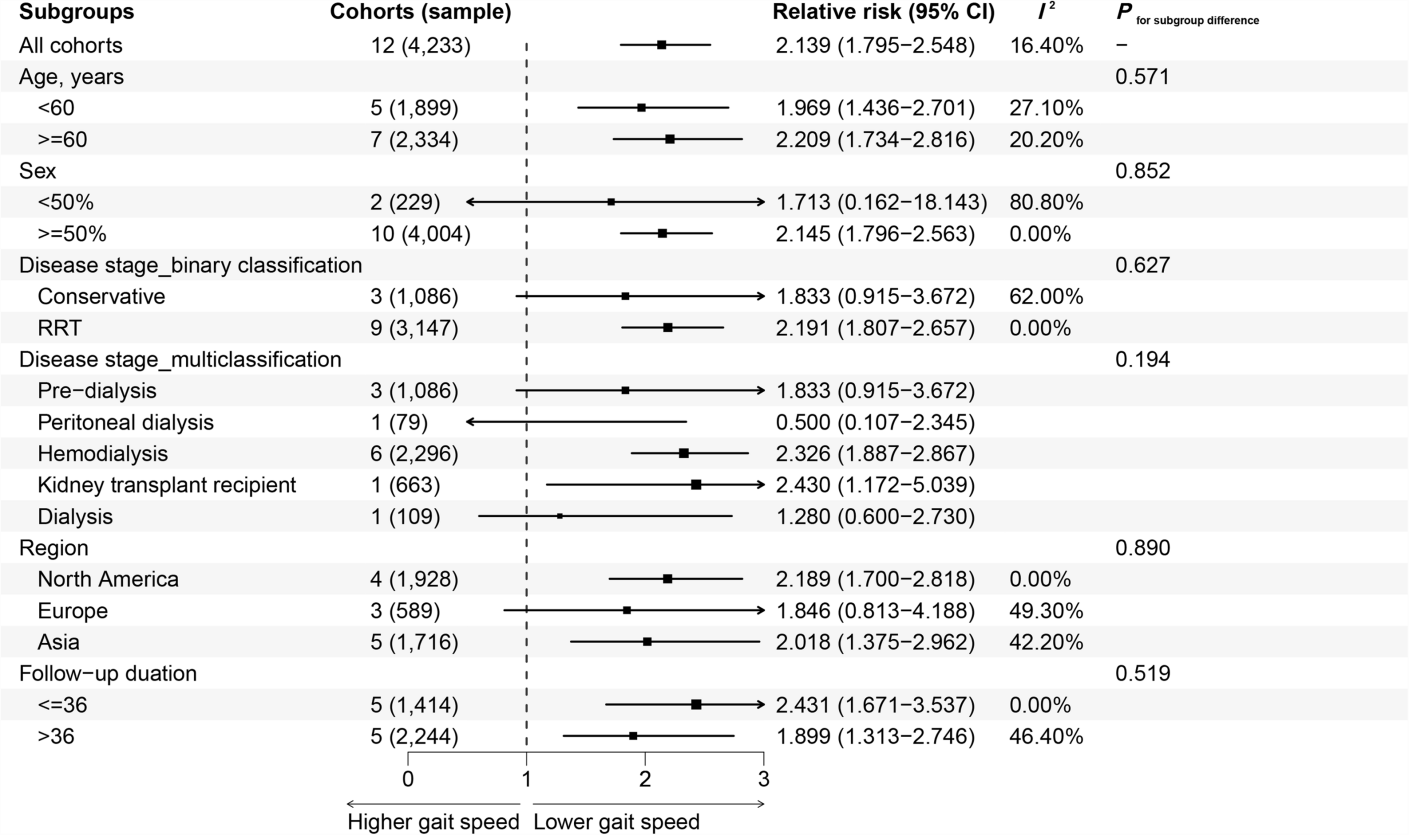
Subgroup analyses of the association between gait speed and all‐mortality risk in CKD patients. Two studies did not report follow‐up times and therefore were not included in subgroup analyses. 95% CI, 95% confidence interval; RRT, renal replacement therapy.

Subgroup analyses were performed for different ages (< 60 vs. > = 60), male predominance (< 50% vs. > = 50%), disease stages (non‐dialysis vs. renal replacement treatment), regions (North America vs. Europe vs. Asia) and follow‐up times (< = 36 vs. > 36 months). Results showed that magnitudes or directions of pooled estimates were robust across subgroups (Figure 3). Meta‐regression results showed that these factors were not a source of heterogeneity in the categorical analysis (Table S6).

In the subgroup analysis by disease stage (multiclassification), the pooled HR for kidney transplant recipients was 2.326 (95% CI: 1.887–2.867), indicating a significant association between slower gait speed and increased all‐cause mortality risk in this subgroup. Compared to other subgroups, such as pre‐dialysis (RR = 2.191; 95% CI: 1.807–2.657) and dialysis (RR = 2.430; 95% CI: 1.172–5.039), kidney transplant recipients demonstrated a similarly strong correlation between gait speed and mortality risk. However, the RR for haemodialysis patients (RR = 0.500; 95% CI: 0.107–2.345) showed a wider confidence interval, likely due to heterogeneity in the included studies.

### 3.5 Dose–Response meta‐Analysis

**FIGURE 4:**
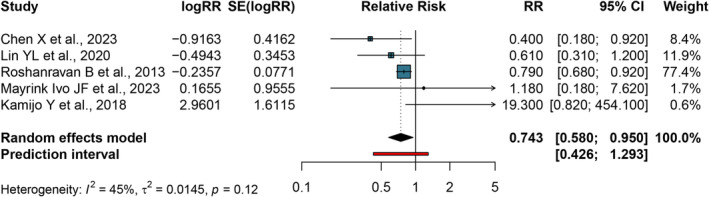
Meta‐analysis of all‐cause mortality per 0.1 m/s increased gait speed. 95% CI, 95% confidence interval; HR, hazard ratio; RR, relative risk.

Figure 4 shows the dose–response analysis of gait speed and all‐cause mortality with 1650 participants and 191 cases. The pooled RR for all‐cause mortality per 0.1 m/s increase in gait speed was 0.743 (95% CI: 0.580–0.955, *I* ^2^ = 45.0%; *P*_for heterogeneity_ = 0.12; 95% prediction intervals: 0.426–1.293). The evidentiary certainty of this result was judged to be high (Table S5).

## 4 Discussion

In this meta‐analysis of prospective cohort studies, we collected available evidence on the association between gait speed and all‐cause mortality risk in adults with CKD. We found from high‐certainty evidence that lower gait speed was associated with a 113.8% increased risk of early death in CKD patients. Each 0.1 m/s increase in gait speed was also associated with a 25.7% lower risk of death. Although the included studies used different walking speed thresholds to define ‘low walking speed’, < 0.8 m/s was the most commonly used. Our sensitivity analysis demonstrated that a walking speed < 0.8 m/s was still associated with a significantly increased risk of death from CKD.

A previous meta‐analysis of six studies (actually five cohorts) showed that lower gait speed was associated with a 145% (95% CI: 1.48–4.08) increased mortality risk in CKD patients compared to higher gait speed [12]. In oncology patients, a meta‐analysis of six cohort studies showed that lower walking speed was associated with a 58% (95% CI: 1.23–2.05) elevated risk of all‐cause mortality compared with higher gait speeds [44]. The MOBILISE‐D study published by Buttery et al. [45] showed that a gait speed of < 0.8 m/s was accompanied by a 255% (95% CI: 1.72–7.36) increased mortality risk in chronic obstructive pulmonary disease compared to a gait speed of > = 0.8 m/s, whereas for each decline in gait speed of 1.0 m/s, there was an increase in mortality risk of up to 655% (95% CI: 1.11–51.3). A recent meta‐analysis of healthy population data that included 50 225 participants showed that gait speed average or brisk/fast was associated with a 20% (95% CI: 12%–28%) and 24% (95% CI: 9%–38%) reduction in risk of all‐cause mortality, compared to self‐reported slow walkers [46]. As a complement, the results of this study provide additional evidence for the health management of CKD patients.

The mechanisms underlying the association between walking speed and premature death risk in CKD patients can be explained in several ways. Most importantly, gait speed captures early multisystem impairments associated with ageing and multimorbidity and is an important marker of functional reserve and resilience [47]. Poor gait speed indicates difficulties in participating in physical activity, and low physical activity levels are associated with an increased risk of early death [48, 49, 50]. Second, a common factor contributing to slow gait speed is the presence of CKD‐related comorbidities (e.g., sarcopenia and diabetes mellitus), which have also been associated with premature death [51]. In addition, slower gait speed is commonly related to endocrine dysfunction (e.g., low testosterone levels), inflammation and oxidative stress, all of which have been linked to a higher risk of mortality in patients with CKD [52]. Finally, faster gait speeds indicate better individual cardiorespiratory fitness, a key predictor of mortality risk in CKD patients [53].

Gait speed is one of the dimensions to assess frailty [54]. Walking speed is the most convenient and useful indicator for mobility. This assessment requires only a timer, and immediate intervention should be undertaken when the measurement falls below the recommended threshold [55]. A recently published study with a large sample showed that physical function declines at different rates as kidney function deteriorates [56], which offers a need for intervention in the early stages of chronic renal failure. The lack of pharmacologic interventions targeting gait decline has led to demands for physical activity and/or exercise to prevent or improve mobility in patients with CKD. A recent systematic review and meta‐analysis showed that a home exercise programme was associated with improved walking speed over a 6‐min walking distance in haemodialysis‐dependent CKD patients [57]—with this in mind, a simple short‐term home exercise prescription, such as lifting free‐weight dumbbells (an item of equal weight can also be used instead) at least three times a week for three sets of 10–15 repetitions each. This approach provides an inexpensive, convenient and safe way to improve gait speed in such a population.

Our subgroup analyses of disease stage suggest significant and plausible effect sizes in renal replacement therapy‐dependent CKD patients and stronger correlations than in conservatively treated individuals. This should be considered when interpreting how gait speed is associated with mortality risk in CKD patients. As kidney function deteriorates, CKD patients are forced to undergo peritoneal dialysis or haemodialysis to prolong their life, a treatment that leads to protein loss and muscle wasting in the body, which are important indications of an impact on gait speed [58]. In contrast, CKD patients who undergo kidney transplantation may obtain an improvement in physical function. Therefore, in future studies, it is necessary to set gait speed thresholds for different CKD stages to observe the correlation between gait speed and mortality risk.

Interestingly, when treated kidney transplant recipients as a distinct category, it revealed a significant association between slower gait speed and increased all‐cause mortality risk in this subgroup (HR = 2.326; 95% CI: 1.887–2.867). This finding underscores the unique clinical characteristics of kidney transplant recipients, who, despite having preserved renal function, may experience muscle function impairments due to long‐term use of immunosuppressants and steroids. These factors likely contribute to the observed relationship between gait speed and mortality risk in this population. Compared to other subgroups, such as pre‐dialysis and dialysis patients, kidney transplant recipients demonstrated a similarly strong correlation between gait speed and mortality risk. This suggests that gait speed is a robust predictor of mortality across different CKD stages, including in populations with improved renal function post‐transplantation. However, the wider confidence intervals observed in the haemodialysis subgroup (HR = 0.500; 95% CI: 0.107–2.345) may reflect heterogeneity, warranting further investigation in future studies.

This study has noteworthy strengths. The meta‐analysis was based on data from prospective cohort studies, a study design that substantially reduces selection and recall bias, but the possibility of reverse causation cannot be completely ruled out. Thus, the pooled results of prospective cohort studies may be the best empirical data to understand the genuine relationship between gait speed and mortality risk in CKD. In addition, most of the included studies had large sample sizes and long follow‐up periods, which helped provide more reliable data for analysis. Our conclusions are strengthened by pooling data from categorical association analyses and dose–response relationship analyses of gait speed and CKD mortality risk.

Several limitations need to be further assessed and considered in future studies when interpreting the results. First, the methods of evaluating gait speed and determining the pace reference group varied across the included studies (including but not limited to 0.8 and 1.0 m/s). These differences may have produced heterogeneity and reduced the precision of the overall association estimates. Nonetheless, sensitivity analyses did not show that any single study's assumptions would significantly impact the association between gait speed and mortality risk in CKD. Second, our findings may be subject to reverse causality bias because participants with faster gait speeds were more likely to engage in more physical activity and have better cardiorespiratory fitness, greater muscle mass and better health status, all of which are key variables influencing the risk of death from CKD; however, only one of the included studies added physical activity as a covariate. Future cohort studies need to consider physical activity variables fully. Third, although the included study statistically adjusted for potential confounders, we could not rule out whether other residual confounders (e.g., lifestyle risk factors, comorbidities or socio‐economic status) might have influenced the observed association between gait speed and mortality. Fourth, our exploratory analyses could not fully account for heterogeneity in the analyses, which may have led to an overestimation of the reported associations. In addition, the small number of studies of pre‐dialysis CKD patients in the present meta‐analysis may limit the interpretation of our results for this subgroup. More studies on pre‐dialysis CKD patients are needed in the future to validate our findings.

Our findings have several important implications for clinical practice. First, gait speed assessment could be a simple, quick and inexpensive tool for risk stratification in CKD patients. Clinicians should consider incorporating gait speed measurement into routine clinical evaluations for CKD patients, as it may help identify those at higher risk of mortality who may benefit from more intensive monitoring and interventions [59]. Second, interventions aimed at improving or maintaining gait speed in CKD patients may have potential benefits for survival. Exercise programmes, particularly those focusing on improving lower extremity strength and function, have shown promise in enhancing gait speed in CKD patients [60]. Third, for patients identified with low gait speed, a comprehensive geriatric assessment might be warranted to identify and address underlying factors contributing to reduced mobility [61]. Finally, gait speed could potentially be used as an outcome measure in clinical trials evaluating interventions aimed at improving physical function and overall health in CKD patients [62].

## 5 Conclusion

This meta‐analysis based on a prospective cohort study showed that lower gait speed was significantly associated with early death in CKD patients, with a 25.7% reduction in the risk of all‐cause mortality for every 0.1 m/s increase in walking speed. As walking is safe and easy to assess and interpret, gait speed can be a significant predictor of death in patients with CKD. To improve the generalizability of these findings, well‐designed large prospective cohort studies are needed to identify age‐, sex‐ and CKD stage‐specific thresholds for walking speed to improve the validity and efficiency of this indicator.

## Author Contributions

F.Z., H.W., Y.B. and Y.F.Z. conceptualized and designed the study, drafted the initial manuscript, coordinated and supervised data collection, analysed the data and reviewed and revised the manuscript. F.Z., Y.B., H.W., L.Y.H. and F.Z. screened articles, abstracted data, drafted the manuscript and reviewed and provided relevant intellectual content. Y.F.Z. and Y.L. revised the manuscript. All authors approved the final manuscript as submitted and agree to be accountable for all aspects of the work.

## Ethics Statement

The authors have nothing to report.

## Conflicts of Interest

The authors declare no conflicts of interest.

## Supporting information


**Table S1** The PRISMA 2020 checklist.
**Table S2** Search detailed for database.
**Table S3** List of studies excluded at full‐text review and reasons for exclusion.
**Table S4** Summary of risk of bias of the included cohort studies.
**Table S5** GRADE evidence profile for overall quality of evidence assessment.
**Table S6** Univariable regression analysis using meta‐regression model‐based on REML.
**Figure S1** Sensitivity analysis of the leave‐one‐out method.
**Figure S2** Funnel plots for gait speed and all‐cause mortality.
**Figure S3** Sensitivity analysis of the leave‐one‐out method (dose–response).
**Figure S4** Sensitivity analysis to a threshold of gait speed for 0.8.

## Data Availability

Data are available upon reasonable request.
