# Supplementary material for: Gait Speed and All‐Cause Mortality in Whole‐Spectrum Chronic Kidney Disease: A Systematic Review and Meta‐Analysis Included 6217 Participants
Source: J Cachexia Sarcopenia Muscle. 2025 Feb 24;16(1):e13739. doi: 10.1002/jcsm.13739 (PMC11848591; doi:10.1002/jcsm.13739)

**Gait speed and all-cause mortality in whole-spectrum chronic kidney disease: a systematic review and meta-analysis**

**Supplementary Material**

**Content**

[Table S1 The PRISMA 2020 Checklist 2](#_Toc180404630)

[Table S2 Search detailed for database. 6](#_Toc180404631)

[Table S3 List of studies excluded at full-text review and reasons for exclusion. 8](#_Toc180404632)

[Table S4 Summary of risk of bias of the included cohort studies. 12](#_Toc180404633)

[Table S5 GRADE evidence profile for overall quality of evidence assessment 13](#_Toc180404634)

[Table S6 Univariable regression analysis using meta-regression model based on REML. 14](#_Toc180404635)

[Figure S1 Sensitivity analysis of the leave-one-out method. 15](#_Toc180404636)

[Figure S2 Funnel plots for gait speed and all-cause mortality. 16](#_Toc180404637)

[Figure S3 Sensitivity analysis of the leave-one-out method (dose-response). 17](#_Toc180404638)

[Figure S4 Sensitivity analysis to a threshold of gait speed for 0.8 18](#_Toc180404639)

Table S1 The PRISMA 2020 Checklist

| **Section and Topic** | **Item #** | **Checklist item** | **Location where item is reported** |
| --- | --- | --- | --- |
| **TITLE** | | |  |
| Title | 1 | Identify the report as a systematic review. | Title page |
| **ABSTRACT** | | |  |
| Abstract | 2 | See the PRISMA 2020 for Abstracts checklist. | Page 2 |
| **INTRODUCTION** | | |  |
| Rationale | 3 | Describe the rationale for the review in the context of existing knowledge. | Page 4 |
| Objectives | 4 | Provide an explicit statement of the objective(s) or question(s) the review addresses. | Page 5 |
| **METHODS** | | |  |
| Eligibility criteria | 5 | Specify the inclusion and exclusion criteria for the review and how studies were grouped for the syntheses. | Page 5-6 |
| Information sources | 6 | Specify all databases, registers, websites, organisations, reference lists and other sources searched or consulted to identify studies. Specify the date when each source was last searched or consulted. | Page 5 |
| Search strategy | 7 | Present the full search strategies for all databases, registers and websites, including any filters and limits used. | Table S2 |
| Selection process | 8 | Specify the methods used to decide whether a study met the inclusion criteria of the review, including how many reviewers screened each record and each report retrieved, whether they worked independently, and if applicable, details of automation tools used in the process. | Page 5 |
| Data collection process | 9 | Specify the methods used to collect data from reports, including how many reviewers collected data from each report, whether they worked independently, any processes for obtaining or confirming data from study investigators, and if applicable, details of automation tools used in the process. | Page 6 |
| Data items | 10a | List and define all outcomes for which data were sought. Specify whether all results that were compatible with each outcome domain in each study were sought (e.g. for all measures, time points, analyses), and if not, the methods used to decide which results to collect. | Page 6 |
|  | 10b | List and define all other variables for which data were sought (e.g. participant and intervention characteristics, funding sources). Describe any assumptions made about any missing or unclear information. | Page 6 |
| Study risk of bias assessment | 11 | Specify the methods used to assess risk of bias in the included studies, including details of the tool(s) used, how many reviewers assessed each study and whether they worked independently, and if applicable, details of automation tools used in the process. | Page 6 |
| Effect measures | 12 | Specify for each outcome the effect measure(s) (e.g. risk ratio, mean difference) used in the synthesis or presentation of results. | Page 7 |
| Synthesis methods | 13a | Describe the processes used to decide which studies were eligible for each synthesis (e.g. tabulating the study intervention characteristics and comparing against the planned groups for each synthesis (item #5)). | Page 6-7 |
|  | 13b | Describe any methods required to prepare the data for presentation or synthesis, such as handling of missing summary statistics, or data conversions. | Page 6-7 |
|  | 13c | Describe any methods used to tabulate or visually display results of individual studies and syntheses. | Page 6-7 |
|  | 13d | Describe any methods used to synthesize results and provide a rationale for the choice(s). If meta-analysis was performed, describe the model(s), method(s) to identify the presence and extent of statistical heterogeneity, and software package(s) used. | Page 6-7 |
|  | 13e | Describe any methods used to explore possible causes of heterogeneity among study results (e.g. subgroup analysis, meta-regression). | Page 6-7 |
|  | 13f | Describe any sensitivity analyses conducted to assess robustness of the synthesized results. | Page 6-7 |
| Reporting bias assessment | 14 | Describe any methods used to assess risk of bias due to missing results in a synthesis (arising from reporting biases). | Page 6-7 |
| Certainty assessment | 15 | Describe any methods used to assess certainty (or confidence) in the body of evidence for an outcome. | Page 7 |
| **RESULTS** | | |  |
| Study selection | 16a | Describe the results of the search and selection process, from the number of records identified in the search to the number of studies included in the review, ideally using a flow diagram. | Figure 1 |
|  | 16b | Cite studies that might appear to meet the inclusion criteria, but which were excluded, and explain why they were excluded. | Table S3 |
| Study characteristics | 17 | Cite each included study and present its characteristics. | Table 1 |
| Risk of bias in studies | 18 | Present assessments of risk of bias for each included study. | Table S4 |
| Results of individual studies | 19 | For all outcomes, present, for each study: (a) summary statistics for each group (where appropriate) and (b) an effect estimate and its precision (e.g. confidence/credible interval), ideally using structured tables or plots. | Page 16-17 |
| Results of syntheses | 20a | For each synthesis, briefly summarise the characteristics and risk of bias among contributing studies. | Page 16-17 |
|  | 20b | Present results of all statistical syntheses conducted. If meta-analysis was done, present for each the summary estimate and its precision (e.g. confidence/credible interval) and measures of statistical heterogeneity. If comparing groups, describe the direction of the effect. | Page 16-17 |
|  | 20c | Present results of all investigations of possible causes of heterogeneity among study results. | Page 16-17 |
|  | 20d | Present results of all sensitivity analyses conducted to assess the robustness of the synthesized results. | Page 16-17 |
| Reporting biases | 21 | Present assessments of risk of bias due to missing results (arising from reporting biases) for each synthesis assessed. | Page 16-17 |
| Certainty of evidence | 22 | Present assessments of certainty (or confidence) in the body of evidence for each outcome assessed. | Table S5 |
| **DISCUSSION** | | |  |
| Discussion | 23a | Provide a general interpretation of the results in the context of other evidence. | Page 18 |
|  | 23b | Discuss any limitations of the evidence included in the review. | Page 20 |
|  | 23c | Discuss any limitations of the review processes used. | Page 20 |
|  | 23d | Discuss implications of the results for practice, policy, and future research. | Page 19 |
| **OTHER INFORMATION** | | |  |
| Registration and protocol | 24a | Provide registration information for the review, including register name and registration number, or state that the review was not registered. | Page 5 |
|  | 24b | Indicate where the review protocol can be accessed, or state that a protocol was not prepared. | Page 5 |
|  | 24c | Describe and explain any amendments to information provided at registration or in the protocol. | Not applicable |
| Support | 25 | Describe sources of financial or non-financial support for the review, and the role of the funders or sponsors in the review. | Page 20 |
| Competing interests | 26 | Declare any competing interests of review authors. | Page 20 |
| Availability of data, code and other materials | 27 | Report which of the following are publicly available and where they can be found: template data collection forms; data extracted from included studies; data used for all analyses; analytic code; any other materials used in the review. | Page 20 |

Table S2 Search detailed for database.

| NO. | Detail | Results |
| --- | --- | --- |
|  | PubMed |  |
| #1 | "Walking Speed"[Mesh] OR "Walking Speeds"[Title/abstract] OR "Walking Speed"[Title/abstract] OR "gait pace"[Title/abstract] OR "Gait Speed"[Title/abstract] OR "Gait Speeds"[Title/abstract] OR "Walking Pace"[Title/abstract] OR "Walking Paces"[Title/abstract] | 17168 |
| #2 | "Renal insufficiency, chronic"[MeSH] OR "Renal Insufficiency"[MeSH] OR "Renal Replacement Therapy"[MeSH] OR "Renal Insufficiency"[Title/Abstract] OR "Kidney Insufficiency"[Title/Abstract] OR "Kidney failure"[Title/Abstract] OR "Renal failure"[Title/Abstract] OR "Kidney disease"[Title/Abstract] OR "Renal disease"[Title/Abstract] OR "Predialysis"[Title/Abstract] OR "Pre-dialysis"[Title/Abstract] OR "End-Stage Kidney"[Title/Abstract] OR "End-Stage Renal"[Title/Abstract] OR "Endstage Kidney"[Title/Abstract] OR "Endstage Renal"[Title/Abstract] OR "Dialysis"[Title/Abstract] OR "Hemodialysis"[Title/Abstract] OR "Haemodialysis"[Title/Abstract] OR "Hemodiafiltration"[Title/Abstract] OR "Haemodiafiltration"[Title/Abstract] OR "Hemofiltration"[Title/Abstract] OR "Haemofiltration"[Title/Abstract] OR "Renal Transplantation"[Title/Abstract] OR "Kidney Grafting"[Title/Abstract] OR "Kidney Transplantation"[Title/Abstract] | 541727 |
| #3 | #1 AND #2 | 301 |
| 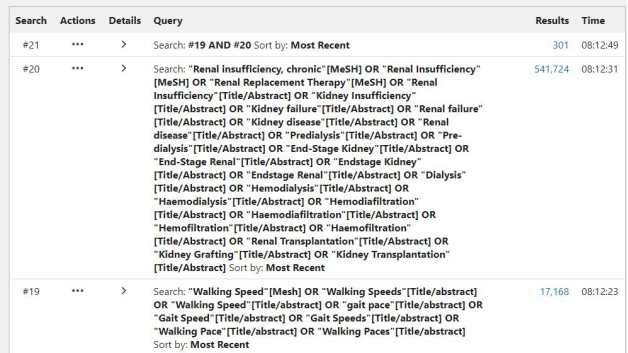 | | |
|  | Embase |  |
| #1 | 'walking speed'/exp OR 'Walking Speeds':ti,ab,kw OR 'Walking Speed':ti,ab,kw OR 'gait pace':ti,ab,kw OR 'Gait Speed':ti,ab,kw OR 'Gait Speeds':ti,ab,kw OR 'Walking Pace':ti,ab,kw OR 'Walking Paces':ti,ab,kw | 32747 |
| #2 | 'kidney failure'/exp OR 'renal replacement therapy'/exp OR 'kidney transplantation'/exp OR 'Renal Replacement Therapy':ti,ab,kw OR 'Renal Insufficiency':ti,ab,kw OR 'Kidney Insufficiency':ti,ab,kw OR 'Kidney failure':ti,ab,kw OR 'Renal failure':ti,ab,kw OR 'Kidney disease':ti,ab,kw OR 'Renal disease':ti,ab,kw OR 'Predialysis':ti,ab,kw OR 'Pre-dialysis':ti,ab,kw OR 'End-Stage Kidney':ti,ab,kw OR 'End-Stage Renal':ti,ab,kw OR 'Endstage Kidney':ti,ab,kw OR 'Endstage Renal':ti,ab,kw OR 'Dialysis':ti,ab,kw OR 'Hemodialysis':ti,ab,kw OR 'Haemodialysis':ti,ab,kw OR 'Hemodiafiltration':ti,ab,kw OR 'Haemodiafiltration':ti,ab,kw OR 'Hemofiltration':ti,ab,kw OR 'Haemofiltration':ti,ab,kw OR 'Renal Transplantation':ti,ab,kw OR 'Kidney Grafting':ti,ab,kw OR 'Kidney Transplantation':ti,ab,kw | 983791 |
| #3 | #1 AND #2 | 779 |
| 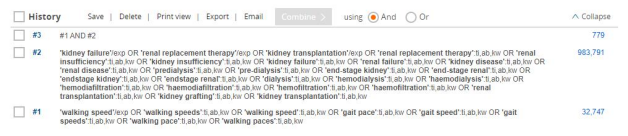 | | |
|  | Web of Science |  |
| #1 | TS=("Walking Speeds" OR "Walking Speed" OR "gait pace" OR "Gait Speed" OR "Gait Speeds" OR "Walking Pace" OR "Walking Paces") | 18502 |
| #2 | TS=("Renal Insufficiency" OR "Kidney Insufficiency" OR "Kidney failure" OR "Renal failure" OR "Kidney disease" OR "Renal disease" OR "Predialysis" OR "Pre-dialysis" OR "End-Stage Kidney" OR "End-Stage Renal" OR "Endstage Kidney" OR "Endstage Renal" OR "Dialysis" OR "Hemodialysis" OR "Haemodialysis" OR "Hemodiafiltration" OR "Haemodiafiltration" OR "Hemofiltration" OR "Haemofiltration" OR "Renal Transplantation" OR "Kidney Grafting" OR "Kidney Transplantation") | 464368 |
| #3 | #1 AND #2 | 304 |
| 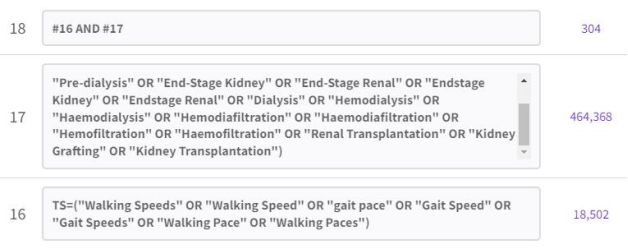 | | |

Table S3 List of studies excluded at full-text review and reasons for exclusion.

| **NO.** | **Reference** | **Reasons** |
| --- | --- | --- |
| 1 | Takahashi R, Yabe H, Ishikawa H, Hibino T, Yamaguchi T, Morishita S, Kono K, Moriyama Y, Yamada T. Age-associated effects of intradialytic exercise on physical function and nutritional status in patients receiving ambulatory hemodialysis: a multicenter cohort study. Hemodial Int. 2023. doi: 10.1111/hdi.13128 | Exposure to non-gait speeds |
| 2 | Kang SH, Do JY, Kim JC. Association between alkaline phosphatase and muscle mass, strength, or physical performance in patients on maintenance hemodialysis. Front Med (Lausanne). 2021;8. doi: 10.3389/fmed.2021.657957 | Exposure to non-gait speeds |
| 3 | Wang C, Guo X, Xu X, Liang S, Wang W, Zhu F, Wang S, Wu J, Zhang L, Sun X, et al. Association between sarcopenia and frailty in elderly patients with chronic kidney disease. Journal of Cachexia, Sarcopenia and Muscle. 2023;14:1855-1864. doi: 10.1002/jcsm.13275 | Exposure to non-gait speeds |
| 4 | Ensrud KE, Lui LY, Cawthon P, Fredman L, Slinin Y, Hillier T, Cauley J, Canales M, Study OF. Cystatin c and objectively measured mobility 10 years later in older women. J Gerontol A Biol Sci Med Sci. 2016;71:1472-1475. doi: 10.1093/gerona/glw037 | Exposure to non-gait speeds |
| 5 | Yabe H, Okada K, Kono K, Imoto Y, Onoyama A, Ito S, Moriyama Y, Kasuga H, Ito Y. Exercise intolerance and malnutrition associated with all-cause mortality in elderly patients undergoing peritoneal dialysis: a single-center prospective cohort study. Int Urol Nephrol. 2023;55:1365-1372. doi: 10.1007/s11255-022-03446-4 | Exposure to non-gait speeds |
| 6 | Hannan M, Chen J, Hsu J, Zhang X, Saunders MR, Brown J, Mcadams-Demarco M, Mohanty MJ, Vyas R, Hajjiri Z, et al. Frailty and cardiovascular outcomes in adults with ckd: findings from the chronic renal insufficiency cohort (cric) study. Am J Kidney Dis. 2023. doi: 10.1053/j.ajkd.2023.06.009 | Exposure to non-gait speeds |
| 7 | Weng SC, Chen YC, Hsu CY, Lin CS, Tarng DC, Lin SY. Impacts of heart failure and physical performance on long-term mortality in old patients with chronic kidney disease. Front Cardiovasc Med. 2021;8. doi: 10.3389/fcvm.2021.680098 | Exposure to non-gait speeds |
| 8 | Imamura K, Yamamoto S, Suzuki Y, Yoshikoshi S, Harada M, Osada S, Kamiya K, Matsuzawa R, Matsunaga A. Prevalence, overlap, and prognostic impact of multiple frailty domains in older patients on hemodialysis. Arch Gerontol Geriatr. 2023;114. doi: 10.1016/j.archger.2023.105082 | Exposure to non-gait speeds |
| 9 | Lorenz EC, Cheville AL, Amer H, Kotajarvi BR, Stegall MD, Petterson TM, Kremers WK, Cosio FG, Lebrasseur NK. Relationship between pre-transplant physical function and outcomes after kidney transplant. Clin Transplant. 2017;31. doi: 10.1111/ctr.12952 | Exposure to non-gait speeds |
| 10 | Wickstrom JF, Sayles HR, Graeff-Armas LA, Yentes JM. The likelihood of self-reporting balance problems in those with advanced chronic kidney disease, slow gait speed, or low vitamin d. Journal of renal nutrition. 2019;29:490-497. doi: 10.1053/j.jrn.2018.10.011 | Exposure to non-gait speeds |
| 11 | Kuki A, Tanaka K, Kushiyama A, Tanaka Y, Motonishi S, Sugano Y, Furuya T, Ozawa T. Association of gait speed and grip strength with risk of cardiovascular events in patients on haemodialysis: a prospective study. BMC Nephrol. 2019;20. doi: 10.1186/s12882-019-1370-6 | Non-death as an outcome |
| 12 | He PP, Ye ZL, Liu MY, Li H, Zhang YY, Zhou C, Wu QM, Zhang YJ, Yang SS, Liu CZ, et al. Association of handgrip strength and/or walking pace with incident chronic kidney disease: a uk biobank observational study. J Cachexia Sarcopenia Muscle. 2023;14:805-814. doi: 10.1002/jcsm.13180 | Non-death as an outcome |
| 13 | Rampersad C, Darcel J, Harasemiw O, Brar RS, Komenda P, Rigatto C, Prasad B, Bohm C, Tangri N. Change in physical activity and function in patients with baseline advanced nondialysis ckd. Clin J Am Soc Nephrol. 2021;16:1805-1812. doi: 10.2215/CJN.07050521 | Non-death as an outcome |
| 14 | Mcadams-Demarco MA, Isaacs K, Darko L, Salter ML, Gupta N, King EA, Walston J, Segev DL. Changes in frailty after kidney transplantation. J Am Geriatr Soc. 2015;63:2152-2157. doi: 10.1111/jgs.13657 | Non-death as an outcome |
| 15 | Fried LF, Lee JS, Shlipak M, Chertow GM, Green C, Ding J, Harris T, Newman AB. Chronic kidney disease and functional limitation in older people: health, aging and body composition study. J Am Geriatr Soc. 2006;54:750-756. doi: 10.1111/j.1532-5415.2006.00727.x | Non-death as an outcome |
| 16 | Liu CK, Lyass A, Massaro JM, D'Agostino RS, Fox CS, Murabito JM. Chronic kidney disease defined by cystatin c predicts mobility disability and changes in gait speed: the framingham offspring study. J Gerontol A Biol Sci Med Sci. 2014;69:301-307. doi: 10.1093/gerona/glt096 | Non-death as an outcome |
| 17 | Roshanravan B, Patel KV, Robinson-Cohen C, De Boer IH, O'Hare AM, Ferrucci L, Himmelfarb J, Kestenbaum B. Creatinine clearance, walking speed, and muscle atrophy: a cohort study. Am J Kidney Dis. 2015;65:737-747. doi: 10.1053/j.ajkd.2014.10.016 | Non-death as an outcome |
| 18 | Kutner NG, Zhang R. Frailty as a dynamic process in a diverse cohort of older persons with dialysis-dependent ckd. Front Nephrol. 2023;3:1031338. doi: 10.3389/fneph.2023.1031338 | Non-death as an outcome |
| 19 | Chiang JM, Kaysen GA, Segal M, Chertow GM, Delgado C, Johansen KL. Low testosterone is associated with frailty, muscle wasting and physical dysfunction among men receiving hemodialysis: a longitudinal analysis. Nephrol Dial Transplant. 2019;34:802-810. doi: 10.1093/ndt/gfy252 | Non-death as an outcome |
| 20 | Moorthi RN, Fadel WF, Cranor A, Hindi J, Avin KG, Lane KA, Thadhani RI, Moe SM. Mobility impairment in patients new to dialysis. Am J Nephrol. 2020;51:705-714. doi: 10.1159/000509225 | Non-death as an outcome |
| 21 | Chen X, Zhu X, Han P, Zhang Y, He M, Zhang Y, Liu J, Tang J, Zhang Y, Zheng Y, et al. Sarcopenia is associated with mild-to-moderate chronic kidney disease in chinese community-dwelling older men but not in women. J Int Med Res. 2022;50. doi: 10.1177/03000605221136683 | Non-death as an outcome |
| 22 | Zemp DD, Giannini O, Quadri P, Rabuffetti M, Tettamanti M, de Bruin ED. Signatures of gait movement variability in ckd patients scheduled for hemodialysis indicate pathological performance before and after hemodialysis: a prospective, observational study. Front Med (Lausanne). 2021;8. doi: 10.3389/fmed.2021.702029 | Non-death as an outcome |
| 23 | Otobe Y, Hiraki K, Hotta C, Izawa KP, Sakurada T, Shibagaki Y. The impact of the combination of kidney and physical function on cognitive decline over 2 years in older adults with pre-dialysis chronic kidney disease. Clin Exp Nephrol. 2019;23:756-762. doi: 10.1007/s10157-019-01698-6 | Non-death as an outcome |
| 24 | Ho JQ, Verghese J, Abramowitz MK. Walking while talking in older adults with chronic kidney disease. Clin J Am Soc Nephrol. 2020;15:665-672. doi: 10.2215/CJN.12401019 | Non-death as an outcome |
| 25 | Harhay MN, Kim Y, Moore K, et al. Modifiable kidney disease risk factors among nondiabetic adults with obesity from the Multi-Ethnic Study of Atherosclerosis. Obesity (Silver Spring). 2023;31(12):3056-3065. doi:10.1002/oby.23883 | Non-death as an outcome |
| 26 | Galen R. Gait and balance in people undergoing long-term hemodialysis. Clin J Am Soc Nephrol. 2023;18:1-2. doi: 10.2215/CJN.0000000000000026 | Letter |
| 27 | Kopple JD. Physical performance and all-cause mortality in ckd. J Am Soc Nephrol. 2013;24:689-690. doi: 10.1681/ASN.2013030307 | Letter |
| 28 | Pham T, Mcneil JJ, Barker AL, Orchard SG, Newman AB, Robb C, Ernst ME, Espinoza S, Woods RL, Nelson MR, et al. Longitudinal association between handgrip strength, gait speed and risk of serious falls in a community-dwelling older population. PLoS One. 2023;18. doi: 10.1371/journal.pone.0285530 | Non-CKD |
| 29 | Sidhu SS, Saggar K, Goyal O, Varshney T, Kishore H, Bansal N, Sidhu SS. Muscle strength and physical performance, rather than muscle mass, correlate with mortality in end-stage liver disease. European Journal of Gastroenterology and Hepatology. 2021;33:555-564. doi: 10.1097/MEG.0000000000001761 | Non-CKD |
| 30 | Jin SH, Park YS, Park YH, Chang HJ, Kim SR. Comparison of gait speed and peripheral nerve function between chronic kidney disease patients with and without diabetes. Ann Rehabil Med. 2017;41:72-79. doi: 10.5535/arm.2017.41.1.72 | Non-cohort study |
| 31 | Johansen KL, Dalrymple LS, Delgado C, Kaysen GA, Kornak J, Grimes B, Chertow GM. Comparison of self-report-based and physical performance-based frailty definitions among patients receiving maintenance hemodialysis. Am J Kidney Dis. 2014;64:600-607. doi: 10.1053/j.ajkd.2014.03.016 | Non-cohort study |
| 32 | Abramowitz MK, Hostetter TH, Melamed ML. Association of serum bicarbonate levels with gait speed and quadriceps strength in older adults. Am J Kidney Dis. 2011;58:29-38. doi: 10.1053/j.ajkd.2010.12.021 | Non-cohort study |
| 33 | Voorend C, Berkhout-Byrne N, Bos WJW, Van Buren M, Mooijaart S. Apathy associates with cognitive dysfunction and mortality in older ckd patients. Nephrol Dial Transplant. 2022;37:i871. doi: 10.1093/ndt/gfac120.003 | Conference abstract |
| 34 | Sheshadri A, Ku E, Adey DB, Lai JC, Johansen KL. Association of physical performance with death or delisting in patients waitlisted for kidney transplantation. J Am Soc Nephrol. 2021;32:632 | Conference abstract |
| 35 | Harhay M, Ranganna K, Michie F, Selinski C, Bajakian T, Levin Mizrahi L, Lee J, Malat G, Xiao G, Reich D, et al. Associations of poor physical and cognitive performance and kidney transplant waiting list denial. Am J Transplant. 2018;18:583-584. doi: 10.1111/ajt.14918 | Conference abstract |
| 36 | Rampersad C, Harasemiw O, Brar RS, Komenda P, Rigatto C, Prasad B, Bohm C, Tangri N. Change in physical activity and function in patients with baseline advanced non-dialysis ckd. J Am Soc Nephrol. 2021;32:287 | Conference abstract |
| 37 | Lorenz E, Cheville A, Kotajarvi B, Cosio F, Lebrasseur N. Change in physical function following kidney transplantation. Am J Transplant. 2015;15 | Conference abstract |
| 38 | Maione J, Salter M, Mcadams Demarco M, Law A, Segev D. Components of frailty and mortality among dialysis patients of all ages. J Am Geriatr Soc. 2014;62:S249. doi: 10.1111/jgs.12870 | Conference abstract |
| 39 | Yabe H, Imoto Y, Ito S, Onoyama A, Okada K, Kasuga H. Exercise tolerance and nutritional status predicts all-cause mortality in older adults on peritoneal dialysis: a single-center prospective observational cohort study. Nephrol Dial Transplant. 2021;36:i399. doi: 10.1093/ndt/gfab101.0015 | Conference abstract |
| 40 | Song Y, Highton PJ, Clarke AL, Burton J, Smith AC. Exploring walking pace, physical activity, and readiness to change in esrd. J Am Soc Nephrol. 2017;28:608 | Conference abstract |
| 41 | Garonzik Wang J, Segev D. Frailty as a novel predictor of delayed graft function in kidney transplant recipients. Transplantation. 2012;94:114 | Conference abstract |
| 42 | Hannan M, Ricardo AC, Brown J, Carmona E, Hajjiri Z, Meza N, Chen J, Saunders MR, Lash JP. Frailty is associated with higher risk of cardiovascular events and death in adults with ckd: findings from the chronic renal insufficiency cohort (cric) study. J Am Soc Nephrol. 2020;31:647 | Conference abstract |
| 43 | Lee YH, Jeong H, Yang DH, Lee SY, Kim JS, Jung SW, Hwang HS, Moon JY, Jeong KH, Lee DY, et al. Gait speed and handgrip strength as predictors of all-cause mortality and cardiovascular events in hemodialysis patients. Nephrol Dial Transplant. 2020;35:i1806. doi: 10.1093/ndt/gfaa142.P1490A | Conference abstract |
| 44 | Liu C, Parvathinathan G, Stedman MR, Seliger SL, Weiner DE, Tamura M. Gait speed and mortality in older adults with ckd: the chronic renal insufficiency cohort. J Am Soc Nephrol. 2023;34:655 | Conference abstract |
| 45 | Beerli N, Gschwind C, Binet I, Dickenmann M, Golshayan D, Hadaya K, Huynh-Do U, Schnyder A, Berres M, De Geest S, et al. Gait speed as single fried frailty phenotype item predictive for length of stay but not readmission in kidney transplantation: a secondary data analysis of a multicenter, prospective cohort study. Swiss Med Wkly. 2021;151:20S-21S | Conference abstract |
| 46 | Ambry S, Moreau K, Juhel F, Merville P, Guerville F, Richert L, Lhomme E, Dehail P. High prevalence of poor locomotor function after kidney transplantation: results of a french prospective study. Transpl Int. 2018;31:23. doi: 10.1111/tri.13101 | Conference abstract |
| 47 | Kutner NG, Zhang R, Huang Y, Painter P. Gait Speed and Mortality, Hospitalization, and Functional Status Change Among Hemodialysis Patients: A US Renal Data System Special Study. Am J Kidney Dis. 2015;66(2):297-304. doi:10.1053/j.ajkd.2015.01.024 | Duplicate cohort |
| 48 | Roshanravan B, Khatri M, Robinson-Cohen C, et al. A prospective study of frailty in nephrology-referred patients with CKD. Am J Kidney Dis. 2012;60(6):912-921. doi:10.1053/j.ajkd.2012.05.017 | Duplicate cohort |

Table S4 Summary of risk of bias of the included cohort studies.

| Author | Representativeness of the exposed cohort | Selection of the non-exposed cohort | Ascertainment of exposure | Demonstration that outcome of interest was not present at start of study | Controls for age or other factors | Ascertainment of outcome | Was follow-up long enough for outcomes to occur (≥36 months) | Adequacy of follow up of cohorts (>80%) |
| --- | --- | --- | --- | --- | --- | --- | --- | --- |
| Mayrink Ivo JF et al., 2023 | ☆ | ★ | ★ | ★ | ☆☆ | ★ | ☆ | ★ |
| Roshanravan B et al., 2013 | ★ | ★ | ★ | ★ | ★★ | ★ | ★ | ★ |
| Clarke AL et al., 2019 | ★ | ★ | ☆ | ★ | ★★ | ★ | ★ | ★ |
| Tabibi H et al, 2020 | ☆ | ★ | ★ | ★ | ★☆ | ★ | ☆ | ★ |
| Nakano Y et al., 2023 | ★ | ★ | ☆ | ★ | ★★ | ★ | ★ | ★ |
| Johansen KL et al., 2019 | ★ | ★ | ★ | ★ | ★★ | ★ | ★ | ★ |
| Lee YH et al., 2020 | ★ | ★ | ★ | ★ | ★★ | ★ | ☆ | ★ |
| Lin YL et al., 2020 | ☆ | ★ | ★ | ★ | ★★ | ★ | ★ | ★ |
| McAdams-DeMarco MA et al., 2017 | ★ | ★ | ★ | ★ | ★★ | ★ | ★ | ★ |
| Chen X et al., 2023 | ★ | ★ | ★ | ★ | ★★ | ★ | ☆ | ★ |
| Nastasi AJ et al., 2018 | ★ | ★ | ★ | ★ | ★★ | ★ | ☆ | ★ |
| Yoshikoshi S et al., 2023 | ★ | ★ | ★ | ★ | ★★ | ★ | ☆ | ★ |
| Kamijo Y et al., 2018 | ☆ | ★ | ★ | ★ | ★★ | ★ | ★ | ★ |
| Sanchez-Tocino ML et al., 2022 | ☆ | ★ | ★ | ★ | ★☆ | ★ | ☆ | ★ |
| Li Y et al., 2021 | ☆ | ★ | ☆ | ★ | ★★ | ★ | ☆ | ★ |
| Brar R et al., 2019 | ☆ | ★ | ★ | ★ | ★★ | ★ | ★ | ★ |
| Yamamoto S et al., 2021 | ★ | ★ | ★ | ★ | ★★ | ★ | ★ | ★ |

Table S5 GRADE evidence profile for overall quality of evidence assessment

| **Certainty assessment** | | | | | | | **№ of patients** | **Effect** | **Certainty** | **Importance** |
| --- | --- | --- | --- | --- | --- | --- | --- | --- | --- | --- |
| **№ of studies** | **Study design** | **Risk of bias** | **Inconsistency** | **Indirectness** | **Imprecision** | **Publication bias** | **[Case/Participants]** | **Relative risk (95% CI)** |  |  |
| **Gait speed and mortality (Categorical analyses)** | | | | | | | | | | |
| 12 | Cohort study | Low risk of bias | Not serious (*I*^2^=16%) | Not serious | Not serious | Not serious | 795/4233 | 2.138 (95% CI 1.794 to 2.548) | ⨁⨁⨁⨁  High | Critical |
| **Gait speed and mortality (Dose-response)** | | | | | | | | | | |
| 5 | Cohort study | Low risk of bias | Not serious (*I*^2^=45%) | Not serious | Not serious | Not applicable | 191/1650 | 0.743 (95% CI 0.580 to 0.950) | ⨁⨁⨁⨁  High | Critical |

Table S6 Univariable regression analysis using meta-regression model based on REML.

| **Variables** | **Estimate** | **SE** | **95% CI** | **z** | ***P*** | ***I*^2^** | **R^2^** |
| --- | --- | --- | --- | --- | --- | --- | --- |
| Age |  |  |  |  |  |  |  |
| Continuous | -0.005 | 0.014 | -0.033, 0.023 | -0.357 | 0.721 | 0.00% | 0.00% |
| Categorical (<60 vs >=60) | 0.068 | 0.179 | -0.283, 0.420 | 0.382 | 0.703 | 0.00% | 0.00% |
| Male |  |  |  |  |  |  |  |
| Continuous | -0.005 | 0.014 | -0.034, 0.023 | -0.382 | 0.703 | 0.00% | 0.00% |
| Categorical (<50% vs >=50%) | 0.111 | 0.532 | -0.931, 1.153 | 0.208 | 0.835 | 0.00% | 0.00% |
| Follow-up |  |  |  |  |  |  |  |
| Continuous | -0.021 | 0.011 | -0.043, 0.001 | -1.840 | 0.066 | 2.72% | 0.00% |
| Categorical (>=36 vs >36) | -0.202 | 0.247 | -0.685, 0.282 | -0.818 | 0.414 | 11.25% | 0.00% |
| Disease stage |  |  |  |  |  | 0.00% | 0.00% |
| Conservative | Ref. |  |  |  |  |  |  |
| RRT | 0.141 | 0.236 | -0.323, 0.604 | 0.595 | 0.552 |  |  |
| Region |  |  |  |  |  | 0.00% | 0.00% |
| Asia | Ref. |  |  |  |  |  |  |
| North America | 0.055 | 0.191 | -0.320, 0.429 | 0.287 | 0.774 |  |  |
| Europe | 0.045 | 0.299 | -0.541, 0.632 | 0.151 | 0.880 |  |  |

RRT, renal replacement therapy; REML, restricted maximum likelihood.

Figure S1 Sensitivity analysis of the leave-one-out method.


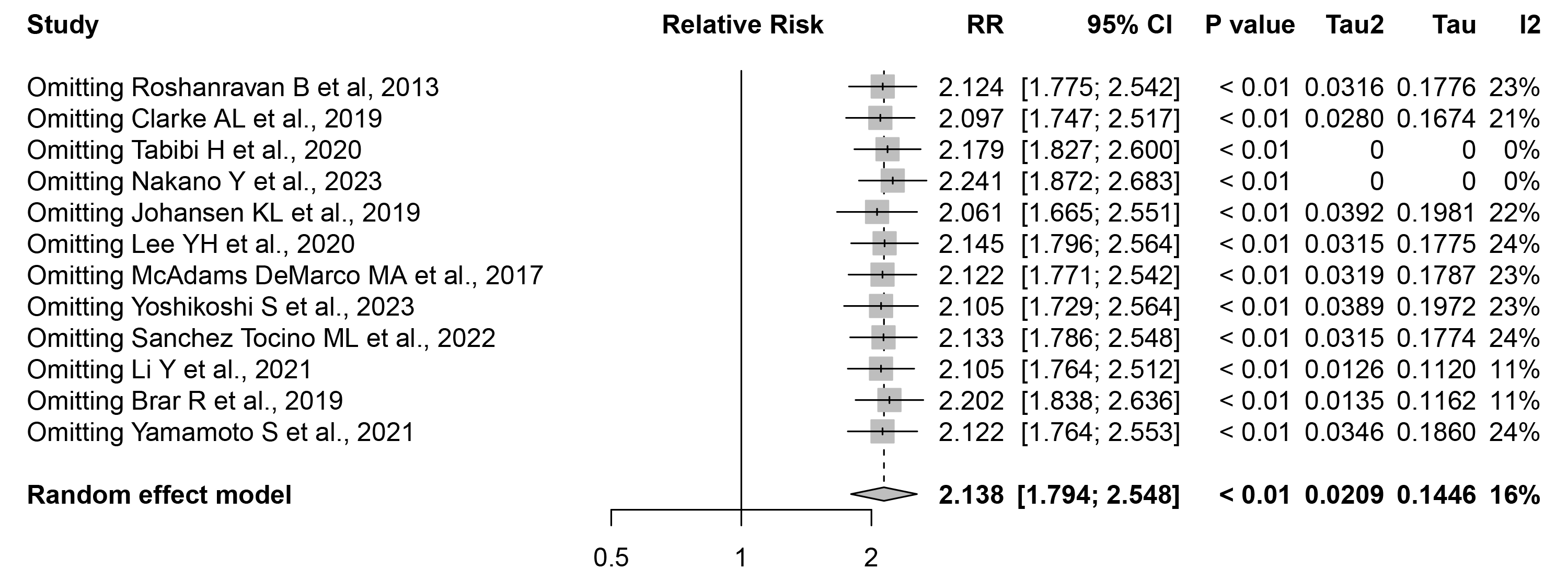


Figure S2 Funnel plots for gait speed and all-cause mortality.


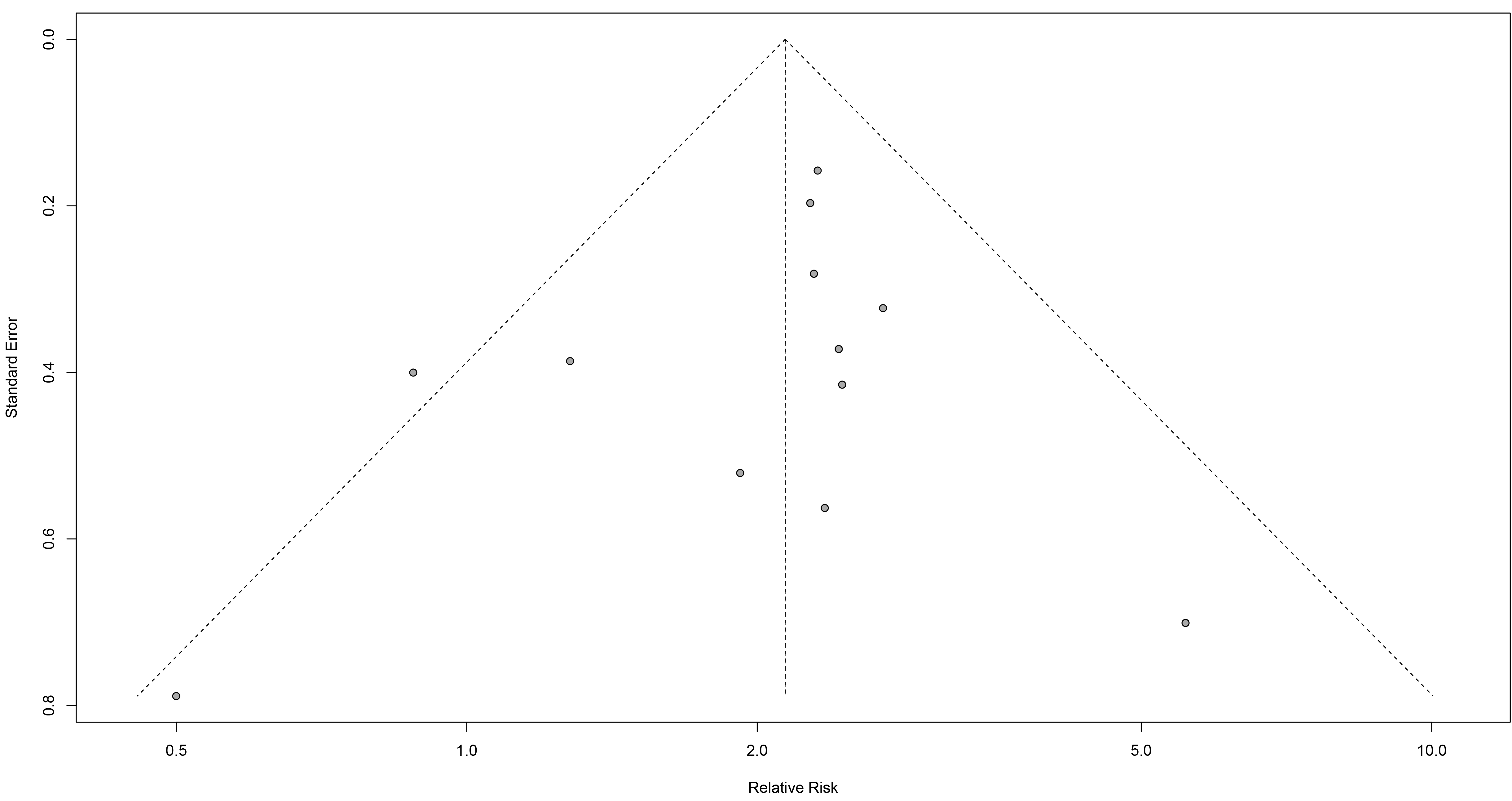


Figure S3 Sensitivity analysis of the leave-one-out method (dose-response).


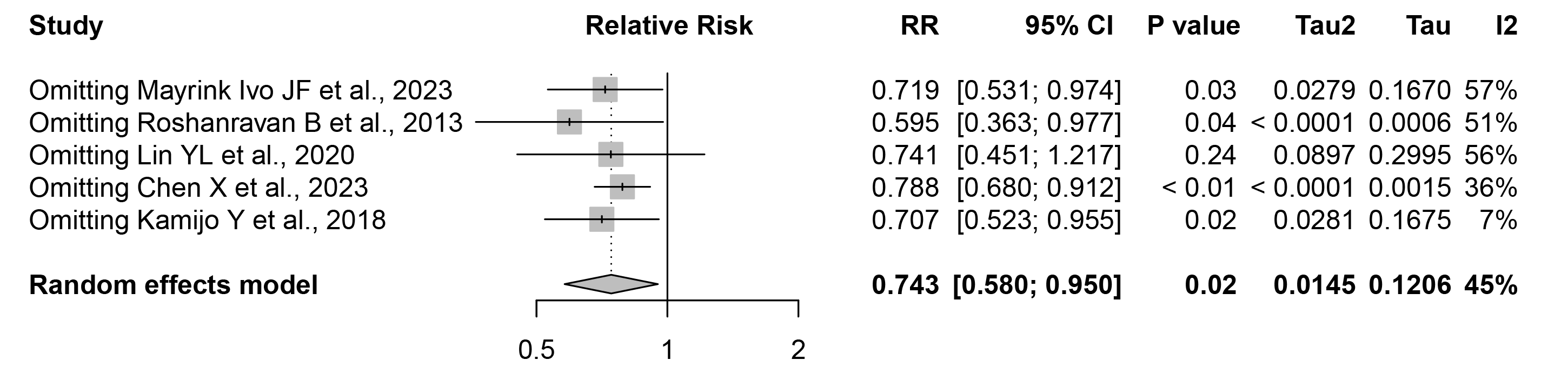


Figure S4 Sensitivity analysis to a threshold of gait speed for 0.8


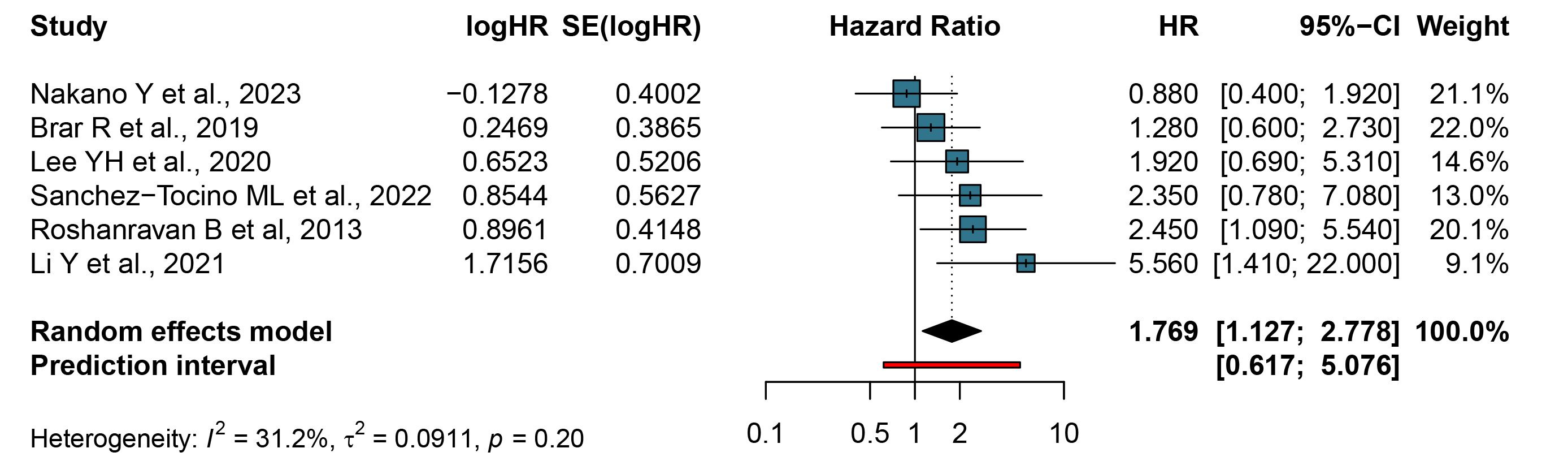

Supplement: Supplementary file 1 — Table S1 The PRISMA 2020 checklist. Table S2 Search detailed for database. Table S3 List of studies excluded at full‐text review and reasons for exclusion. Table S4 Summary of risk of bias of the included cohort studies. Table S5 GRADE evidence profile for overall quality of evidence assessment. Table S6 Univariable regression analysis using meta‐regression model‐based on REML. Figure S1 Sensitivity analysis of the leave‐one‐out method. Figure S2 Funnel plots for gait speed and all‐cause mortality. Figure S3 Sensitivity analysis of the leave‐one‐out method (dose–response). Figure S4 Sensitivity analysis to a threshold of gait speed for 0.8. [file JCSM-16-e13739-s001.docx]
